# *Giardia intestinalis* and Fructose Malabsorption: A Frequent Association

**DOI:** 10.3390/nu11122973

**Published:** 2019-12-05

**Authors:** María Trelis, Silvia Taroncher-Ferrer, Mónica Gozalbo, Vicente Ortiz, José M. Soriano, Antonio Osuna, Juan F. Merino-Torres

**Affiliations:** 1 Area of Parasitology, Department of Pharmacy and Pharmaceutical Technology and Parasitology, University of Valencia, 46010 Valencia, Spain; silvia.taroncher@fundacions.uv.es; 2 Joint Research Unit on Endocrinology, Nutrition and Clinical Dietetics, University of Valencia-Health Research Institute La Fe, 46026 Valencia, Spain; jose.soriano@uv.es (J.M.S.); merino_jfr@gva.es (J.F.M.-T.); 3 University Clinic of Nutrition, Physical Activity and Physiotherapy (CUNAFF), Lluís Alcanyís Foundation-University of Valencia, 46020 Valencia, Spain; 4 Area of Nutrition and Bromatology, University of Valencia, 46010 Valencia, Spain; monica.gozalbo@uv.es; 5 Department of Gastroenterology, University and Polytechnic Hospital La Fe, 46026 Valencia, Spain; ortiz_vicbel@gva.es; 6 Food & Health Lab, Institute of Materials Science, University of Valencia, 46980 Paterna, Spain; 7 Biochemistry and Molecular Parasitology Group, Department of Parasitology, Institute of Biotechnology, University of Granada, 18003 Granada, Spain; aosuna@ugr.es; 8 Department of Endocrinology and Nutrition, University and Polytechnic Hospital La Fe, 46026 Valencia, Spain; 9 Department of Medicine, University of Valencia, 46010 Valencia, Spain

**Keywords:** parasites, *Giardia intestinalis*, *Blastocystis* sp., carbohydrates, malabsorption, fructose, risk factors

## Abstract

Nowadays, scientific studies are emerging on the possible etiological role of intestinal parasites in functional digestive disorders. Our study was carried out with healthy individuals (control group; *n* = 82) and symptomatic patients with lactose or fructose malabsorption, including positive (malabsorbers; *n* = 213) and negative (absorbers; *n* = 56) breath test, being analyzed for the presence of intestinal parasites. A high parasitic prevalence was observed in malabsorbers (41.8%), exclusively due to single-cell eukaryotes but not helminths. *Giardia intestinalis* was the predominant parasite in cases of abnormal absorption (26.5%), significantly associated with fructose malabsorption and doubling the probability of developing this pathology. Within controls, *Blastocystis* sp. (13.4%) was almost the only parasite, being the second among patients (12.6%), and *Cryptosporidium parvum*, the last species of clinical relevance, was detected exclusively in two malabsorbers (0.9%). The consumption of ecological food and professions with direct contact with humans arose as risk factors of parasitism. A diagnosis of carbohydrate malabsorption in adulthood is the starting point, making the search for the primary cause necessary. Accurate parasitological diagnosis should be considered another tool in the clinical routine for patients with recurrent symptoms, since their condition may be reversible with adequate therapeutic intervention.

## 1. Introduction

Parasitic diseases have an enormous impact on human and animal health. Among them, intestinal parasites affect a large proportion of the world’s population, both in rural and urban areas [1,2]. Children are particularly susceptible to parasitic infections and to the development of an acute symptomatology; but, in adults, chronic courses can be asymptomatic or present as nonspecific mild symptoms that result in a low clinical index of suspicion for the diagnosis [3,4].

Increasingly, scientific studies are emerging on the possible etiological role of intestinal parasites in functional digestive disorders, characterized by abdominal discomfort associated with an altered intestinal reactivity in response to luminal (infectious agents or food) or psychological stimuli. Several authors have agreed on the positive correlation between pathologies (such as dyspepsia, carbohydrate intolerance/malabsorption, food intolerance, and irritable bowel syndrome (IBS)) and intestinal protozoal diseases highlighting *Giardia intestinalis* [5,6,7,8].

*G. intestinalis* causes around 280 million human cases of diarrhea every year and infects more than 40 animal species [9,10,11]. It is the etiological agent of most diarrhea outbreaks caused by contaminated water, making it a water-borne disease [12]. The role of contaminated food in the spread of giardiasis is not well-documented, but it is thought that 7%‒15% of infections are acquired through food transmission, making it a water- and food-borne disease [11,13]. In high-income countries, a prevalence of 2%‒7% is estimated, while in low-income countries with tropical/subtropical climates and deficient hygienic-sanitary conditions, a level of infection of 40% or even higher can be reached in children [14,15]. The clinical spectrum of giardiasis in humans is wide-ranging, from asymptomatic cases to acute diarrhea and malabsorption syndromes [16,17] such as lipid- and fat-soluble vitamin malabsorption, and also vitamin B12 deficiency [5,18], leading to rapid and acute weight loss, compromising child development and the work capacity of adults.

Giardiasis appears due to the noninvasive colonization of the upper part of the small intestine, duodenum, and jejunum. The direct and indirect pathogenic action of the protozoan as well as the inflammatory response activated in the host cause damage to the absorptive mucosa, which may be the origin of enterocyte apoptosis and accelerated cell turnover, villus shortening, a reduction in disaccharide activity, a loss of barrier function, and the penetration of commensal bacteria [19,20,21,22]. This set of pathophysiological actions provokes alterations in the digestion, with the absorption of nutrients triggering the characteristic symptoms of giardiasis such as diarrhea, abdominal distension and pain, loss of appetite, flatulence, and rapid weight loss, respectively.

The traditional diagnosis of *Giardia* is based on the light microscopic observation of cysts in stool samples previously treated by concentration methods. Trophozoites or cysts can also be observed in stained fecal samples (trichrome, iron hematoxylin) or by direct immunofluorescence using monoclonal antibodies for the detection of antigens [23]. However, since cysts do not appear regularly in feces, the diagnostic effectiveness of coprology, within a non-personalized health system, is ineffective, leaving many cases undiagnosed. The elimination of the cysts follows three patterns of excretion: (a) high excretion, where cysts are found in all the stool samples of the patient; (b) low excretion, where cysts appear in approximately 30% of the samples; and (c) a mixed pattern of excretion, where the parasites appear every one to three weeks with high excretion after a period of low excretion, due to variations in the population present in the intestine as a consequence of the immunological pressure and the antigenic variations of the parasite. As a consequence of the intermittent nature of the excretion of the cysts, the sensitivity of a stool test with a single stool sample is at most 40%. Concentration techniques and the study of three samples, preferably obtained every other day, increase the sensitivity to 85% [24]. In patients with chronic diarrhea and malabsorption syndromes, in whom a coprological examination leads to repeated negative results, the microscopic search for trophozoites in the duodenal fluid or a duodenal biopsy could be useful [25]. 

An alternative method to light microscopy is the detection of *Giardia* antigens in the stool by immunochromatography assays (ICA); these are considered simple, specific, and sensitive. Currently, there are also commercialized enzyme immunoassays (EIA) that use monoclonal antibodies against parasite antigens, with a specificity of 99% (99.3% to 100%) and sensitivity in the range of 88.6%–100%. The usefulness of EIA of the serum for the diagnosis of human giardiasis is controversial. The specificity and sensitivity mainly depend on the type of antigen used, the immunoglobulin studied, and the prevalence of infection in the geographical area. In fact, the existence of significant differences in serological IgG antibody levels between individuals with symptomatic and asymptomatic giardiasis has not been demonstrated. Nevertheless, the presence of secretory IgA (sIgA) antibodies has been detected in the saliva of individuals with active giardiasis, recognizing a series of native antigens of the parasite [26,27,28]. Everything indicates that sIgA present in the saliva can be used as a marker of infection, with the advantage of being elevated while the infection by the protozoan is active and disappearing a short time after its elimination; moreover, the sampling is noninvasive [29].

On the other hand, carbohydrates contribute, in the Western diet, around 50%‒55% of the total energy consumed in a day. Disaccharides (sucrose, lactose, maltose, isomaltose, and trehalose) make up an important proportion of the daily intake, requiring a process of hydrolysis by specific enzymes located in the intestinal microvilli to be absorbed. As a result of this enzymatic activity, monosaccharides such as glucose, galactose, and fructose are released and absorbed using transporters located in the apical and basolateral membranes of the enterocyte [30,31,32]. In that case, non-absorbed carbohydrates will provide fermentable substrate to the large intestine bacteria and increase osmolality in the lumen, thus causing the development of gastrointestinal disorders with the characteristic symptoms. The clinical manifestations will begin between 30 min and 2 h after the ingestion of the sugars [33,34]. The prevalence of carbohydrate malabsorption in the general population is not well-documented. In studies with patients with gastrointestinal symptoms, 60% were registered for fructose, 51% for lactose, and 33% for both [35,36]. In fact, lactase (β-galactosidase) has to hydrolyze this sugar in the small intestine before the complete absorption of the derivate monosaccharaides by the transmembrane transporters (SLGT-1, GLUT-5, and GLUT-2) and its activity in the human small intestine has been described from the jejunum to the proximal ileum [37]. For fructose, it is rapidly absorbed into the bloodstream by active transporters GLUT-5 and GLUT-2, present in the first portion of the small intestine, being the degree of malabsorption depends not only on the number of competent transporters, but also on the quantity and quality of the mixture of sugars present in the intestinal lumen [32,37]. 

Nowadays, the “gold standard” method for the diagnosis of carbohydrate malabsorption is the noninvasive “breath test”, in which the concentration of hydrogen (H_2_) and methane (CH_4_) in the exhaled air is measured after the ingestion of and overload of sugar as a substrate. Those gases are produced by the bacterial activity in the colon when fermenting sugars that have not been properly absorbed in the small intestine [38,39,40]. Currently, the treatment of patients with abnormal absorption of sugars in the diet consists of the long-term exclusion of monosaccharides and disaccharides, with a resulting impact on their diet, health status, and quality of life. The finding of intestinal parasites could, thus, drastically change the course of this disease, from being considered chronic to having a specific treatment aimed at curing the parasitic disease and, consequently, the malabsorption syndrome. The associated deficits are bound to cease when the intestinal function and the patient´s food consumption normalize. In some patients with malabsorption, dietary management with restrictions on dietary sugars does not provide a noticeable improvement, so other primary causes for the underlying disorders are suspected, requiring studies from another perspective, such as misdiagnosed intestinal parasitism. The aim of this study was to evaluate the presence of intestinal parasites (with an emphasis on *G. intestinalis*) in patients with persistent gastrointestinal symptoms and fructose or lactose malabsorption in order to select the most effective diagnostic strategy and to perform an adequate therapeutic intervention.

## 2. Materials and Methods

### 2.1. Patients and Controls

A total of 351 individuals were enrolled. Two hundred and sixty-nine patients (60 male, 209 female; median age 44 years) with at least two of the following symptoms: abdominal pain, bloating or flatulence, and altered bowel habits, who attended the Gastroenterology Department at La Fe University Hospital to be evaluated for carbohydrate malabsorption (lactose and fructose) by H_2_/CH_4_ breath tests from November 2013 to December 2016. Eighty-two healthy individuals (24 male, 58 female; median age 38 years), recruited as controls, were patients without gastrointestinal disorders and with a negative breath test. Patients with digestive bleeding, neoplastic antecedents, antibiotic treatment in the last 30 days, and chronic treatments with nonsteroidal anti-inflammatory drugs were excluded from the study.

As shown in Table 1, among the symptomatic patients, 213 had a positive breath test and constituted a subgroup called “Malabsorbers” (41 male, 172 female; median age of 40.5 years), who were, therefore, referred to the Endocrinology and Nutrition Department for nutritional management and parasitological examinations. Fructose malabsorption was most frequent in 80.3% of cases, followed by 59.6% in the case of lactose. In addition, 39.4% of patients were positive for both sugars. The remaining 56 symptomatic patients who had negative tests were classified as “Absorbers” (19 male, 37 female; median age of 47.4 years). Detailed information of relevant metadata per patient is shown in Table S1.

Clinical and epidemiological data were obtained from each subject included in the study using standardized questionnaires that covered aspects such as the personal evaluation of the median degree of abdominal distension and flatulence by means of a visual analogue scale (VAS) (from 0 to 10) [41], the results of breath tests, and comorbidities related to chronic gastrointestinal disorders (Table 1). Regarding gastrointestinal symptoms, a predominance of diarrhea was recorded more frequently in malabsorbers. Likewise, self-referred abdominal distension and flatulence reached slightly higher scores in the malabsorption subgroup. Finally, with the data available in the medical records about chronic intestinal pathologies such as irritable bowel syndrome (IBS), inflammatory bowel diseases (IBD), and celiac diseases (CD), considered as comorbidities, information was completed. Patients suffering from carbohydrate malabsorption presented varied gastrointestinal comorbidities in the form of IBS (10.3%), CD (2.8%), and IBD (2.4%), while in absorbers a high percentage of IBS (39.2%) was detected. Furthermore, patients and controls underwent routine blood analyses.

During the interview upon admission, the participants were also asked about certain risk factors, personal situations and habits, potentially related to the acquisition of intestinal parasites. The parameters analyzed were compiled from epidemiological studies on intestinal parasites in industrialized countries [12,42] and were as follows: (1) nationality of risk: people who, although permanently residing in Spain, originate from endemic countries and who travel regularly to those countries; (2) profession of risk: jobs with direct contact with humans; (3) consumption of ecological fruit or vegetables: this kind of farming avoids the use of synthetic chemicals and sometimes uses feces as fertilizer; (4) contact with animals: pets or other animals for hobbies or for combining rural and urban life and; (5) trips to endemic countries in the last five years.

### 2.2. Parasitological Examination

Three stool samples taken on alternate days were obtained from each individual. The first two samples were collected in the REAL Mini System® with SAF (Durviz®, Valencia, Spain) containers prelabelled and designed for preservation and concentration by centrifugation in one step. The third sample was collected fresh in an empty container without any fixative and a small amount was used for the simultaneous qualitative detection of *Cryptosporidium parvum*, *Giardia intestinalis*, and the *Entamoeba histolytica* complex antigens by immunochromatography assay (ICA) (Simple *Crypto*-*Giardia-Entamoeba*®, Operon®, Zaragoza, Spain); the rest of the sample was then fixed like the other two samples to continue with the analyses. All three samples were concentrated and examined by light microscopy for the detection of intestinal parasites in general, protozoan cysts, and intestinal helminths eggs.

For the specific diagnosis of *G. intestinalis,* two coprological tests for the detection of the parasite (light microscopy and commercial immunochromatography assay) and an enzyme immunoassay (EIA) for host-specific antibodies (anti-*Giardia* secretory IgA (sIgA)) were used. The combination of several methods is especially recommended in the case of adults with chronic infections in whom the elimination of cysts to the environment may be irregular, scarce, or null.

A saliva sample from each individual was collected by placing a small sterile cotton ball under the tongue in contact with the oral mucosa for 10 min. Thereafter, the cotton was introduced to a microtube with a preservative medium consisting of 195 μL of protease inhibitor (2×) (Complete Mini EDTA-free^®^, Roche, Basel, Switzerland) and 3 μL of bacteriostatic (ProClin 300^®^, Sigma Aldrich, St. Louis, MO, USA), and preserved at 4 °C. Saliva (400–500 µL) was recovered from the cotton by compression with a syringe and then centrifuged at 10,300 *g* for 10 min to discard debris; the resulting supernatant was collected and kept at −80 °C until use. An “in-house” indirect ELISA for the detection of specific salivary IgA antibodies to *G. intestinalis* was developed following the protocols of previous studies [29,43] with some modifications. Total trophozoite antigen was obtained as previously described by Hassan et al. (2002) [27] from in vitro axenic cultures (strains ATCC 30888, ATCC 30957 and ATCC 50137).

*G. intestinalis* antigen diluted in 0.1 M carbonate‒bicarbonate buffer (pH 9.6) (10 µg/well) was incubated overnight at 4 °C. Excess of antigen was washed off with PBS-Tween (0.05%) (PBST) and blocked for 1 h at 37 °C with bovine serum albumin (BSA) diluted to 0.5% in PBS (PBS-BSA). Samples diluted 1:2 in PBS-BSA were dispensed in duplicate (100 µL/well) and incubated for 90 min at 37 °C. After three washes with PBST, an incubation with the secondary antibody, peroxidase-conjugated anti-human IgA (goat polyclonal anti-human IgA alpha chain, Abcam, Cambridge, MA, USA), in a dilution of 1:10,000 in PBS-BSA was made for 1 h at 37 °C. Wells were washed three times with PBST and substrate solution (0.04% of ortho-phenylenediamine (OPD) and 0.001% of hydrogen peroxide in 0.05 phosphate (0.2 M)‒citrate (0.1 M) buffer, pH 5.0 (Sigma Aldrich)) was added (100 µL/well) for 10 min in the darkness and the reaction stopped with HCl (3N) (100 µL/well). The optical density (OD) was measured at 490 nm by an iMark Microplate Absorbance Reader (Bio-Rad Laboratories, Hercules, CA, USA). The cutoff values were measured as mean OD readings of negative controls ± 3 standard deviations (SD).

### 2.3. Analytical Determinations

With blood samples in hand, full hematology and biochemistry profiles were made for each participant, and, in addition, the levels of some vitamins (A, E, D, B12, and folic acid) and minerals (Mg, Fe, P) along with the value of serum albumin and anemia markers (hemoglobin, hematocrit and, red blood cells counts) were requested in order to evaluate nutritional disorders associated with malabsorption syndromes. In the same way as markers of inflammation or immune response activation, serum levels of immunoglobulin (IgA, IgM, IgG, IgE), ferritin, C-reactive protein, and leukocyte counts were determined.

### 2.4. Statistical Analysis

The analytical data for the descriptive analysis were summarized by means and standard deviation or median, together with first and third quartiles in the case of continuous variables. For the analysis of categorical variables (presence of parasites, analytical alterations, etc.), absolute and relative frequencies were calculated. 

To study the association between the presence of intestinal parasites and variables such as gender, age, study group, type of carbohydrate malabsorption, and parasitic risk factors, logistic regression models were used. From these models, the odds ratio (OR), adjusted for the presence of the parasite, as well as an estimation of 95% confidence intervals were obtained. In this sense, to determine the association between the presence of parasites and analytical alterations in markers of absorption (vitamins and minerals) and markers of infection, multiple linear regression models were used; *p*-values below 0.05 were considered significant in all cases.

All analyses were performed using R software (version 3.4.3; R Foundation for Statistical Computing, Vienna, Austria) and the ordinal R packages (version 2018.8-25), NMF (version 0.21), clickR (version 0.4.05), and nlme (version 3.1-135), with the support of the Biostatistics and Bioinformatics Department at Health Research Institute La Fe.

### 2.5. Ethical Statement

Informed consent was obtained from all participants after being informed about the aim of the study, risks, and implications of their participation in it, as well as the treatment and confidentiality of the data. 

This study was approved by the Biomedical Research Ethics Committee of University and Polytechnic Hospital La Fe in 07/04/2015, respecting the fundamental principles of the Declaration of Helsinki, of the Council of Europe Convention in relation to Human Rights and Biomedicine of the UNESCO Declaration.

## 3. Results

### 3.1. Presence of Intestinal Parasites

Table 2 shows the presence of intestinal parasites between patients (42.0%) and controls (14.6%); exclusively intestinal single-cell parasites and no helminths were diagnosed. *G. intestinalis* was diagnosed, with a combination of tests, in 24.5% of the symptomatic patients (13 male, 56 female; median age 38 years). In healthy individuals, the predominant and almost exclusive species was *Blastocystis* sp. (13.4%), detected by light microscopy.

Cases of multiparasitism were detected in 6.3% of the studied population. The most frequent parasitic co-infection was *G. intestinalis* and *Blastocystis* sp. in nine cases (3.3%). Two cases of *Cryptosporidium parvum* (0.7%) were also detected in association with *G. intestinalis* by fecal immunochromatography assay. The intestinal amoebas *Endolimax nana*, *Entamoeba coli*, *Entamoeba hartmanni*, and *Iodamoeba *buetschlii** were identified by light microscopy and considered of less clinical relevance; all of them occurred in the form of multiparasitism, double and triple, in symptomatic individuals.

The distribution of cases of parasitism was analyzed according to gender and age. Although the sample is not balanced in terms of gender, due to a greater influx of female patients to the gastroenterology department, the value is practically the same in the genders: 34.5% for females and 32.1% for males.

If the frequency of parasitization within symptomatic patients is analyzed, the global prevalence of parasitization in cases of malabsorbers (41.8%) and absorbers (42.9%) are very close, although far from the healthy controls (14.6%). What is also noteworthy is the fact that the predominant species varies depending on the group and subgroup. Among the 213 patients with malabsorption, *G. intestinalis* predominated with 26.3%, exceeding that detected in absorbers (17.9%), and far above that of healthy individuals (1.2%), resulting in statistically significant differences compared with the controls (*χ*^2^ = 19.7; *p* ˂ 0.001) (Table 2).

The second species detected among patients was *Blastocystis* sp., without significant differences between groups, malabsorbers (13.6%), absorbers (8.9%), and healthy individuals (13.4%). *C. parvum*, the last species of clinical relevance, was detected exclusively in two malabsorbers (0.9%) associated with *G. intestinalis* and together with an immunopathology, namely selective IgA deficiency.

### 3.2. Comparison of the Effectiveness of the Methods Used for G. intestinalis Diagnosis

In order to optimize the diagnosis of *G. intestinalis*, considering that patients were adults with chronic gastrointestinal problems, added to the intermittence in the elimination of parasitic forms to the environment for this parasite, direct and indirect methods were combined to try to improve the sensitivity and avoid false negatives. A patient was considered a positive case if one of the tests applied resulted in a positive outcome.

A commercial immunochromatography assay (ICA) for the detection of *G. intestinalis* antigens in fresh stool samples was performed in each participant, and 10 positive cases were identified (2.8%) (Table 3). After examining three concentrated stool samples per participant by light microscopy, another seven positive (4.8%) cases were added. All those positive cases detected by ICA were also confirmed by microscopy.

To complete the diagnosis, direct methods were combined with an indirect enzyme-linked immunosorbent assay (ELISA) for the detection of specific sIgA anti-*Giardia* antibodies in saliva samples. In the saliva, sIgA is elevated while the infection is active, and its concentration is not subject to periods of intermittency. By means of indirect ELISA, a total of 62 positive cases were detected, increasing the value to 17.7% (Table 3). All positive cases detected by ICA or microscopy coincided with positive sIgA values, although a high proportion of patients with direct negative tests and positive antibodies results was also encountered.

### 3.3. Analytical Changes in the Study Population

Blood examination of patients and controls did not show remarkable hematological or biochemical abnormalities. No differences were found in the markers of anemia (hemoglobin, hematocrit, and red blood cells count), in the levels of minerals, or in the total antibody levels, respectively, in leukocyte counts.

**Figure 1:**
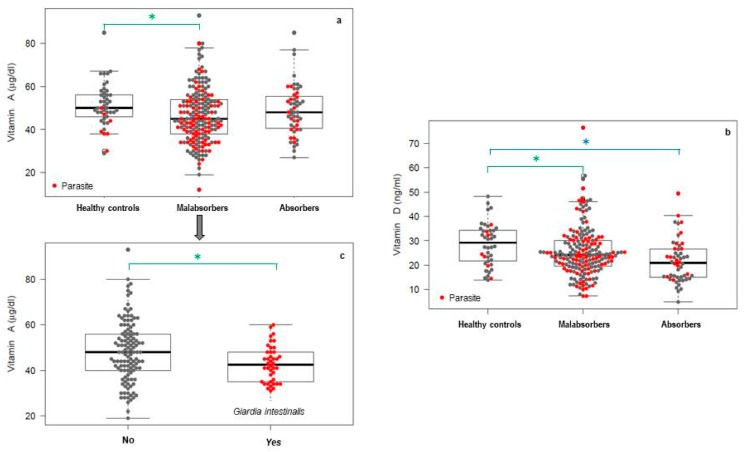
Swarm plot of levels of (**a**) vitamin A, (**b**) vitamin D in the study group, and (**c**) *G. intestinalis* and vitamin A in studied malabsorbers. * Statistically significant (*p* < 0.05) respect to control.

Nevertheless, in the case of fat-soluble vitamins, more or less marked deficit situations were detected depending on the vitamin and study group. Vitamin A levels of the symptomatic group were lower than the controls and even lower in the case of malabsorbers, 40% of whom were below the reference range. When the presence of *G. intestinalis* was added, the frequency of vitamin A deficit reached 51.6%. The comparison by linear regression of the vitamin A levels between groups is shown in Figure 1 for comparisons that were statistically significant. Malabsorbers’ levels of vitamin A were 4.70% below those of healthy controls, a result with statistical significance (Figure 1a). Among subjects with absorption failure, those with giardiasis showed significantly lower levels of vitamin A than noninfected subjects, with values being 6.64% lower (Figure 1b). In the case of *Blastocystis* sp., although the average value for vitamin A was also lower in parasitized malabsorbers, the difference was not marked enough to be significant.

In the case of vitamin D, a situation of generalized deficiency when compared with the reference range was detected, even in the healthy population. Individuals with gastrointestinal symptomatology (malabsorbers and absorbers) showed significant reduction in the levels of vitamin D in comparison with control (Figure 1c), but did not show differences significantly levels of vitamin D between malabsorbers non-parasitized and with giardiasis (data not reflected in Figure 1). The existence of a negative effect on vitamin D levels and the presence of intestinal parasites among patients with malabsorption could not be demonstrated due to the general deficit observed.

### 3.4. Potential Risk Factors for Intestinal Parasites

The frequency of the association between harboring intestinal parasites and each habit and living condition considered as a potential risk factor was calculated and analyzed by multivariable logistic regression (Table 4). The dependent variable was a positive diagnosis of intestinal parasites, while the independent variables were nationality of risk, profession of risk, contact with animals, regular consumption of ecological food, and having travelled to endemic countries in the last five years.

Multivariate analysis of risk factors detected that the consumption of ecological vegetables, and fruit especially, was the strongest predictor of intestinal parasites among the participants (Table 4). In addition, professions involving direct contact with humans or travel to endemic areas led to a significant risk of infection. Having contact with animals or having a nationality of risk were not significantly associated with parasitism, although those in question showed a certain predisposing tendency with an association frequency greater than 40%.

### 3.5. Analysis of the Association of Intestinal Parasites and Carbohydrate Malabsorption

Based on a presence of intestinal parasites of 41.8% among malabsorbers, an initial analysis of the association of both variables was performed. The likelihood of developing absorption failure is higher in the case of parasitism (OR = 2.27; CI 95% = 1.39‒3.78; *p* ˂ 0.001).

Fructose malabsorption was the most frequent syndrome (63.6%). In this context, the probability of this abnormal absorption was analyzed according to the presence or absence of intestinal parasites. In relation to “upper small intestine” parasites, i.e., *G. intestinalis*, 89.5% of the parasitized individuals were unable to absorb fructose (Table 5). Logistic regression confirmed that the presence of *G. intestinalis* often manifests as inability to absorb fructose, with a significant association (Table 5). The role of *Blastocystis* sp., a colonic parasite, in fructose assimilation was also assessed. Of the 45 patients diagnosed with this parasite, 51.1% suffered from fructose malabsorption. However, despite this high percentage of association, a significant association was not confirmed after statistical analysis (Table 5), probably due to the high frequency of this parasite among the control population. Furthermore, the relative risk posed by *Giardia* was estimated at 2.0, which is interpreted as the presence of this protozoan doubling the likelihood of displaying this secondary syndrome, which can be considered harmful.

The probability of suffering lactose malabsorption among parasitized cases (37%) was slightly above that in non-parasitized cases (32%). In addition, as shown in Table 5, 50% of cases of this kind of malabsorption were detected among those parasitized by *Giardia*. Statistical analysis showed no significant relationship for this type of abnormality in absorption and the species that affect the duodenum/jejunum. In a final analysis, 42.2% of patients parasitized with *Blastocystis* sp. presented lactose malabsorption, but a statistical significance was not found in this case either (Table 5).

## 4. Discussion

In the investigations conducted by Grazioli et al., in Italy (2006) [6], the prevalence of *G. intestinalis* in their symptomatic patients with irritable bowel syndrome and dyspepsia was determined by a combination of direct techniques, light microscopy, and a commercial sandwich ELISA applied to two types of samples, feces, and duodenal aspirate, obtaining a total prevalence of 6.5%. In the same way, *G. intestinalis* determination in feces and duodenal aspirate by Fouad et al. [4] in 2014 in Cairo, Egypt, also including patients with dyspepsia, showed frequencies of giardiasis lower than those obtained in this work. They obtained 10% positivity by light microscopy, and with conventional PCR, the number of positives increased to 15.8%. It should be noted that the diagnostic methods employed search for parasitic forms or its DNA, but do not assess the response to infection developed in the host. In relation to our results, it is important to highlight that this parasite was only detected in one of the control individuals (1.2%). Thus, it can be affirmed that, in our population, the presence of *G. intestinalis* is usually related to gastrointestinal symptomatology, even extraintestinal, despite the fact that the possibility of asymptomatic cases is always considered in the existing literature. A study of *Giardia* prevalence conducted by Abulhasan et al. (2013) [44] among patients with dyspepsia obtained 11% prevalence by light microscopy of feces and 19% by capture ELISA for parasitic fecal antigens. Perhaps this higher prevalence was not conditioned by the type of sample or diagnostic technique, but by the nationality of the participants, since they all resided in an area endemic for giardiasis, Egypt, with a prevalence of 44% in dyspeptic patients found by the analysis of duodenal biopsies and feces when examining by light microscopy [25]. As noted in previous studies, and confirmed here, an alternative to traditional coprological analysis in giardiasis is the detection of secretory IgA in saliva. Those studies demonstrated a correlation between the presence of elevated saliva sIgA levels and active intestinal infection, and can therefore be considered a useful tool for monitoring giardiasis and evaluating treatment efficacy. The elimination of the parasite will cause a rapid decrease in salivary levels of this immunoglobulin, unlike what occurs with levels of IgG in serum that remain elevated for months or even years after parasite elimination [29]. On the other hand, the detection of serum monomeric IgA proved to be significantly less sensitive than the detection of sIgA in saliva [29,43,45]. *G. intestinalis*, though a noninvasive protozoan, is capable of stimulating lymphoid organs and provoking a specific mucosal response that can be used for indirect diagnosis when conventional coprology analyses might be negative, but symptoms persist. Although little is known about possible extraintestinal locations of *Giardia* (the bile or a pancreatic duct) [46], it has been suggested that before an immune response, the number of intestinal protozoan decreases, and escaping trophozoites will lodge in the pancreatic or biliary duct while they change their antigenic cover (variable surface proteins, VSP) as a mechanism of immune evasion. Thereafter, they might periodically colonize the mucosa of the duodenum, to which they will resort for feeding and replication. In these periods of intestinal absence, stool cysts would not be eliminated and direct diagnosis would lead to false negatives, even using the most sensitive of direct methods. Hence, thus far, there is still no “gold standard” available for the diagnosis of giardiasis. Therefore, diagnostic sensitivity is improved by combining a diagnostic method that assesses the parasite intestinal presence with another that assesses the host response to the infection. Once the prevalence of intestinal parasites between the study population and their absorption status was determined, the probability of both situations coexisting was analyzed. A significant relationship between the variables was confirmed, indicating a high probability that individuals with intestinal parasites will suffer some kind of malabsorption, or, analyzed in reverse, patients with malabsorption might present intestinal parasitism (around 40%). This situation had already been cited in previous studies such as that carried out by Moya-Camarena et al. (2002) [5], concluding that an effect on mucosal absorption may be one of the intestinal side effects generated by intestinal parasites. Of the total cases of malabsorption, a predominance of the inability to absorb fructose was observed (63.6%), and this condition was studied in relation to the presence or absence of intestinal parasites. In the case of *G. intestinalis* residing in the upper part of the small intestine, a significant relationship between its presence and the development of this kind of malabsorption was detected, allowing for its classification as harmful to the host and posing a double risk of developing such a syndrome. The association between giardiasis and a decrease in disaccharidase activity (lactase) had already been cited previously, but this effect of parasitization on the absorption of monosaccharaides, fructose in particular, has, so far, not been studied or cited. The link between the factors might be conditioned by the fact that they involve the same intestinal region [32], so the parasite and the associated inflammatory response trigger fructose malabsorption due to a decrease in the activity of specific transporters. In the case of lactose malabsorbers, a significant relationship with this protozoan could not be demonstrated, although the frequency of the association was high. In this sense, the aforementioned secondary lactose malabsorption in cases of *G. intestinalis* cannot be corroborated [6], probably due to the large number of non-parasitized lactose malabsorbers, as a consequence of nonparasitic pathologies or even since they had developed malabsorption through the voluntary reduction of the consumption of milk and milk products.

For *Blastocystis* sp., a higher prevalence in patients with irritable bowel syndrome than in controls was detected previously [47,48]. The relationship between *Blastocystis* sp. and the gastrointestinal symptoms and malabsorption cannot be well defined with the present results. In a recent prospective study of 3070 immigrants in El Ejido (Almería, Spain), attending a Tropical Medicine Unit with gastrointestinal symptoms, without more specificity, 570 cases of blastocystosis (18.5%) were diagnosed. These results are close to those of our study, despite the fact that the diagnostic method was light microscopy and the study population also consisted of adults. This high prevalence is likely due to the fact that the patients analyzed mostly came from sub-Saharan Africa and the Maghreb, where hygienic standards are not comparable to those in Spain [49]. The fact that *Blastocystis* sp. has such a high prevalence in the control population could cause doubts about its pathogenic potential. There might be genetic differences at the subtype level, giving it a greater or lesser pathogenicity [50] or, as suggested in other studies, its virulence may be related not so much to its genetics as to the host intestinal micro-environment, as determined by the host immune status and its interplay with the microbiota or co-infections with other pathogens [51]. A brief comment on *Blastocystis* sp. (colonic parasite) is required given its high prevalence among patients. A significant relationship between its presence, in the large intestine, and carbohydrate malabsorption, either of lactose or fructose, was established. Its pathogenic role is still controversial, and, consistent with our results, it has been identified in both symptomatic and asymptomatic subjects. The latest studies suggest that many external factors such as resident microbiota or host immune status determine the virulence or pathogenicity of the parasite [51]. Its presence may only be circumstantial, i.e., the cause is some kind of alteration in the intestinal immune system due to another primary cause and *Blastocystis* has taken advantage of it to settle and aggravate the situation of imbalance.

On the other hand, the total annual cases of cryptosporidiosis declared in Spain according to data from the National Centre of Epidemiology, Health Institute Carlos III (2016) [52] was 557, compared to 2969 of giardiasis, which, epidemiologically, is much less widespread. It is noteworthy that the two cases of *C. parvum* included in our study turned out to be subjects with a selective IgA deficit, which predisposes them to the acquisition of mucosal infections.

Upon admission to the study, all participants underwent a blood test to assess levels of some vitamins and minerals, albumin and anemia indicators (hemoglobin, hematocrit, and red blood cells counts) in relation to intestinal absorption, as well as markers of infection/inflammation such as immunoglobulin, C-reactive protein, ferritin, and leukocyte count. One of the main results was the detection of lower average values of vitamin A in the group of patients with respect to the control group, although in both cases within the reference range established for this variable. This could be related to the fact that all patients suffer from gastrointestinal symptoms, diarrhea, and associated nutrient malabsorption syndromes, which was expected and consistent with previous studies [53,54]. When vitamin A deficiencies were analyzed based on patient subgroups, a prevalence of 40% in patients with positive breath tests was observed. In that subgroup of patients, mucosal integrity is more compromised, and carbohydrate failure of absorption is part of a general malabsorption syndrome with loss of all nutrients including proteins, lipids, and fat-soluble vitamins [55,56,57,58]. In addition, as a consequence of hypovitaminosis A, intestinal defense is compromised and a greater predisposition to pathogen infections in the gastrointestinal tract is acquired [59]. Regarding the possible effect of a specific parasite, caused by the parasitic habitat or its pathogenic potential, on vitamin A levels, it is noteworthy that 51.6% of individuals affected by *G. intestinalis* presented a deficit, with a statistically significant association, in accordance with previous studies about local effects of giardiasis [21,55,56,57,60,61]. Giardiasis causes a shortening of the villi and microvilli of the small intestine, as well as a decrease in the barrier effect and absorption capacity [56]. Its direct consequence is nutrient malabsorption, since this fat-soluble vitamin is absorbed in the upper part of the small intestine, specifically in the duodenum, a place of adhesion and the parasitic habitat of *G. intestinalis* [53]. 

The present results on vitamin D levels were unexpected since the analytics showed a generalized deficit situation in all the study groups. Therefore, it could not be demonstrated that the presence of the parasite had an effect on the absorption of vitamin D, although the deficit was more pronounced in patients. The main sources of vitamin D for humans are synthesis after sun exposure and some foods such as blue fish [56]. In healthy individuals, levels would be expected to be within the reference range, since they do not have any gastrointestinal pathology that interferes with absorption and, living on the coast of Spain, they benefit from a climate in which sun exposure is usual, which should a priori be a positive factor in the absorption of this vitamin. Recent epidemiological studies support a state of vitamin D deficiency or insufficiency in the population of almost the entire world, partly related to the photoprotection measures promoted to prevent skin cancer. On the other hand, the latest recommendations of the Institute of American Medicine consider that serum levels of 20 ng/mL seem sufficient and attainable for the general population, even under conditions of minimal sun exposure. Taking into account the previous considerations, the prsevalence of this almost pandemic hypovitaminosis D reported in recent years could be overestimated [62]. It would therefore be interesting to perform an analytical assessment of vitamins with the aim of minimizing or alleviating the deficits caused by parasites and malabsorption, and promoting self-defense and the recovery of the intestinal mucosa.

Other participant data of interest were those related to their habits, customs or personal situation that involved a risk of acquisition of parasites in the environment. The variables analyzed were taken from previous epidemiological studies on parasitic diseases in high-income countries [12,42]. Among the parameters considered, three intestinal parasites predictors were detected. The habit that stood out above the others, being identified in a large proportion of cases of parasitism, was the increasing consumption of ecological food, especially fruit and vegetables. This relationship was so significant that it can be considered the main cause of infection among the study population. The other two relevant risk factors were having a profession that involves direct contact with humans and having travelled to endemic areas in recent years. Vegetables and fruit grown through ecological production are usually fertilized with animal droppings or irrigated with water that could be contaminated with human or animal feces. Subjects who consume raw fruit and vegetables (lettuce, tomatoes, etc.) and do not adequately wash these foods with food bleach could increase their risk of infection [63]. Some authors have already suggested that ecological food is involved in the alimentary transmission of intestinal parasites due to fecal contamination [11,12,64]. Thus, special attention should be paid to the risk factors described, such as increased travel to tropical and subtropical countries in recent years and especially the consumption of ecological fruit and vegetables, in order to avoid future infections or reinfections.

## 5. Conclusions

The parasitization has been found to be higher than expected in symptomatic patients and still higher in cases of carbohydrate malabsorption, although comparable studies are scarce. *G. intestinalis* is the most prevalent parasite in the symptomatic patient groups, followed by *Blastocystis* sp., coinciding with the data obtained for the general adult population in high-income countries. A combination of direct and indirect diagnostic techniques is essential to optimize the diagnosis of giardiasis among adults and immunocompetent subjects, and is generally helpful in chronic cases. 

*G. intestinalis* may be the origin of fructose malabsorption given its significant degree of coexistence. *G. intestinalis* infection and the malabsorption syndromes that frequently accompany it are responsible for deficits of fat-soluble vitamins, most markedly in the case of vitamin A, and, therefore, nutritional measures must be implemented together with the recommended drug treatment to achieve earlier clinical improvement and minimize its nutritional impact. 

Accurate parasitological analysis, including nonroutine molecular methods, is essential for the effective treatment of carbohydrate malabsorption and should be considered another clinical tool for use with adult patients with long-term gastrointestinal symptoms after discounting other possible causes, since only with nutritional management can significant improvement be achieved. Secondary malabsorption of carbohydrates is of multifactorial origin and the role played by parasites therein remains to be studied, since they may act as triggers, as conditioning or as aggravating factors of the disease.

## Figures and Tables

**Table 1:** Characteristics at baseline of the patients enrolled in the study.

	Malabsorbers (213)	Absorbers (56)
Gender		
Female, *n* (%)	172 (80.8%)	37 (66.1%)
Male, *n* (%)	41 (19.2%)	19 (33.9%)
Age		
Years (mean)	40.5	47.4
minimum‒maximum	29‒55	30‒64
Type of malabsorption		
Fructose, *n* (%)	171 (80.3%)	
Lactose, *n* (%)	127 (59.6%)	
Combined, *n* (%)	84 (39.4%)	
Gastrointestinal symptoms		
Diarrhea, *n* (%)	204 (95.8%)	50 (89.3%)
Constipation, *n* (%)	112 (52.6%)	22 (39.3%)
Abdominal distension (VAS > 5), *n* (%)	197 (92.5%)	49 (87.5%)
Flatulence (VAS > 5), *n* (%)	203 (95.3%)	51 (91.1%)
Comorbidities		
Irritable Bowel Syndrome, *n* (%)	22 (10.3%)	7 (39.2%)
Inflammatory Bowel Disease, *n* (%)	5 (2.4%)	
Celiac Disease, *n* (%)	6 (2.8%)	

*n* = number of cases; % = relative frequency; VAS: visual analogue scale.

**Table 2:** Presence of intestinal parasites depending on the group of study.

Intestinal Parasites	Patients (269)				Grouped Patients (269)		Controls (82)	
	Malabsorbers (213)		Absorbers (56)					
	*n*	%	*n*	%	*n*	%	*n*	%
Giardia intestinalis ^#^ *	56	26.3 *	10	17.9 *	66	24.5	1	1.2
*Blastocystis* sp.	29	13.6	5	8.9	34	12.6	11	13.4
*Endolimax nana*	1	0.5	2	3.6	3	1.1	0	0.0
*Entamoeba coli*	0	0.0	3	5.4	3	1.1	0	0.0
*Entamoeba hartmanni*	1	0.5	3	5.4	4	1.1	0	0.0
*Cryptosporidium parvum*	2	0.9	0	0.0	2	0.7	0	0.0
*Iodamoeba buetschlii*	0	0.0	1	1.8	1	0.4	0	0.0
Total	89	41.8	24	42.9	113	42.0	12	14.6

*n* = number of cases; % = relative frequency; ^#^ Combination of diagnostic methods; * Statistically significant (*p* ˂ 0.05) respect to control.

**Table 3:** Laboratory test for *G. intestinalis* detection in the study population (*n* = 351).

	Feces		Saliva
	Immunochromatography	Light Microscopy	Indirect ELISA
Positive (69)	10 (2.8%)	17 (4.8%)	62 (17.7%)
Negative (282)	341 (97.2%)	334 (95.2%)	289 (82.3%)

**Table 4:** Analysis of predictors of intestinal parasites in the study population.

Variables	*N*	With IP		OR	95% CI	*p* Value
		*n*	%			
Nationality of risk	17	9	52.9	1.32	0.41, 4.29	0.640
Profession of risk	56	26	46.4	2.16	1.14, 4.21	0.022 *
Contact with animals	89	37	41.6	1.67	0.95, 2.92	0.072
Regular consumption of ecological food	180	95	52.8	6.81	4.03, 11.9	0.001 *
Having travelled to endemic countries in the last five years	75	37	49.3	1.90	1.03, 3.57	0.040 *

IP: Intestinal Parasites; *N*: *n* of individuals that registered this variable; *n*: *n* of individuals with IP and this variable; OR: Odds Ratio; CI: Confidence Interval; * significant association (*p* ˂ 0.05).

**Table 5:** Distribution of specific carbohydrate malabsorption among *G. intestinalis* and *Blastocystis* sp. cases.

Type of Malabsorption	*G. intestinalis*				*Blastocystis* sp.			
	Association	OR	95% CI	*p* Value	Association	OR	95% CI	*p* Value
Fructose	89.5%	3.21	1.8‒5.7	˂0.001 *	51.1%	0.85	0.4‒1.6	0.076
Lactose	50.0%	1.23	0.7‒2.3	0.089	42.2%	1.24	0.6‒2.4	0.099

OR: odds ratio; CI: confidence interval; * significant association (*p* ˂ 0.05).

## Supplementary Materials

The following are available online at https://www.mdpi.com/2072-6643/11/12/2973/s1, Table S1: Detailed information of relevant metadata per patient.
